# Group schema therapy versus group cognitive behavioral therapy for social anxiety disorder with comorbid avoidant personality disorder: study protocol for a randomized controlled trial

**DOI:** 10.1186/s13063-016-1605-9

**Published:** 2016-10-08

**Authors:** Astrid Baljé, Anja Greeven, Anne van Giezen, Kees Korrelboom, Arnoud Arntz, Philip Spinhoven

**Affiliations:** 1 Department of Anxiety, PsyQ, Lijnbaan 4, 2512 VA The Hague, The Netherlands; 2 Institute of Psychology, Section of Clinical Psychology, Leiden University, Wassenaarseweg 52, 2333 AK Leiden, The Netherlands; 3 Department of Clinical Psychology, University of Amsterdam, PO Box 15933, 1001 NK Amsterdam, The Netherlands; 4 Department of Medical and Clinical Psychology Tilburg University, PO Box 90153, 5000 LE Tilburg, The Netherlands

**Keywords:** Social anxiety disorder, Randomized controlled trial, Group schema therapy, Group cognitive behavioral therapy, Avoidant personality disorder

## Abstract

**Background:**

Social anxiety disorder (SAD) with comorbid avoidant personality disorder (APD) has a high prevalence and is associated with serious psychosocial problems and high societal costs. When patients suffer from both SAD and APD, the Dutch multidisciplinary guidelines for personality disorders advise offering prolonged cognitive behavioral therapy (CBT). Recently there is increasing evidence for the effectiveness of schema therapy (ST) for personality disorders such as borderline personality disorder and cluster C personality disorders. Since ST addresses underlying personality characteristics and maladaptive coping strategies developed in childhood, this treatment might be particularly effective for patients with SAD and comorbid APD. To our knowledge, there are no studies comparing CBT with ST in this particular group of patients. This superiority trial aims at comparing the effectiveness of these treatments. As an additional goal, predictors and underlying mechanisms of change will be explored.

**Methods/design:**

The design of the study is a multicentre two-group randomized controlled trial (RCT) in which the treatment effect of group cognitive behavioral therapy (GCBT) will be compared to that of group schema therapy (GST) in a semi-open group format. A total of 128 patients aged 18–65 years old will be enrolled. Patients will receive 30 sessions of GCBT or GST during a period of approximately 9 months. Primary outcome measures are the Liebowitz Social Anxiety Scale Self-Report (LSAS-SR) for social anxiety disorder and the newly developed Avoidant Personality Disorder Severity Index (AVPDSI) for avoidant personality disorder. Secondary outcome measures are the MINI section SAD, the SCID-II section APD, the Schema Mode Inventory (SMI-2), the Inventory of Depressive Symptomatology Self-Report (IDS-SR), the World Health Organization Quality of Life-BREF (WHOQOL-BREF), the Difficulties in Emotion Regulation Scale (DERS), the Rosenberg Self-Esteem Scale (RSES) and the Acceptance and Action Questionnaire (AAQ-II). Data will be collected at the start, halfway and at the end of the treatment, followed by measurements at 3, 6 and 12 months post-treatment.

**Discussion:**

The trial will increase our knowledge on the effectiveness and applicability of both treatment modalities for patients suffering from both diagnoses.

**Trial registration:**

Dutch Trial Register: NTR3921. Registered on 25 March 2013.

**Electronic supplementary material:**

The online version of this article (doi:10.1186/s13063-016-1605-9) contains supplementary material, which is available to authorized users.

## Background

Beginning with the Diagnostic and Statistical Manual of Mental Disorders (DSM)-III [1] and continuing in DSM-IV [2], individuals whose fears are manifest in most social situations are assigned to the generalized subtype of social anxiety disorder (SAD), while individuals whose fears are more circumscribed are grouped together as a separate category, referred to as non-generalized social anxiety disorder. Since the introduction of the generalized subtype, there is a controversy about the differences with avoidant personality disorder (APD) [3]. While some researchers emphasize that APD is a serious form of generalized SAD [4, 5], a growing number of studies indicate that there is a qualitative difference between the two disorders. Shortcomings in establishing interpersonal relationships and severe feelings of inferiority are seen as cardinal features of APD [3, 6, 7].

To preserve continuity with clinical practice, the categorical diagnoses and criteria for personality disorders in the DSM-5 are kept the same. An alternative dimensional model is added in which, besides limitations in (inter)personal functioning, specific maladaptive traits pertaining to the dimensions of ‘detachment’ and ‘negative affectivity’ characterize persons with APD. Detachment is reflected in maladaptive traits such as withdrawal, anhedonia and intimacy avoidance, while anxiousness and worry in relation to social situations characterize these patients with respect to negative affectivity [8]. Furthermore, on the basis of empirical findings, it has been suggested that avoidance is a dominant coping strategy not only in social but also in non-social situations in APD [9–11].

APD is associated with high societal costs due to frequent use of somatic and mental health care, a high risk for developing other mental disorders and suboptimal professional functioning. Furthermore, patients report a low quality of life, and for family members, having a relative with a diagnosis of APD is often a considerable burden [12].

In clinical practice, there is no consensus about which treatment is indicated for patients with diagnoses of both SAD and APD. In the Netherlands the multidisciplinary guidelines recommend offering prolonged cognitive behavioral therapy (CBT) in the case of SAD with comorbid APD [13].

A small number of effectiveness studies have shown that CBT and pharmacological interventions are effective for patients with SAD and comorbid APD [14, 15]. Research among a sample of patients with social anxiety and patients with social anxiety and comorbid APD showed that APD was not predictive of CBT treatment outcome, and that several subjects who received a diagnosis of APD before treatment no longer met criteria for APD after treatment [16]. CBT in group format is approximately as effective as individual CBT [17].

Furthermore, there is growing evidence that schema therapy (ST) is an effective treatment for patients with APD [18, 19]. Research has shown that APD is associated with emotional neglect and abuse in the past [20, 21]. When the normal, healthy developmental needs of childhood are not met, maladaptive schemas develop. Activation of these schemas can trigger an emotional, cognitive and behavioral state, which in ST is called a ‘schema mode’ [22]. APD can be well conceptualized in terms of schema modes, with detached and avoidant protector modes as prominent coping modes, lonely child and abandoned/abused child mode as prominent child modes and punitive parent mode as prominent internalized parent mode [10, 23]. Within ST different techniques are applied, including experiential techniques such as imagery rescripting and mode role-plays, that explicitly address dysfunctional coping modes. A new development is group ST (GST), where specific methods and techniques are applied to use the group process in order to facilitate the process of change [22, 24].

Because of the promising results of ST, we designed a superiority trial with the primary objective of investigating the effectiveness of group schema therapy (GST) compared to prolonged group CBT (GCBT) for patients with social anxiety disorder (SAD) and comorbid avoidant personality disorder (APD). More specifically the following research question has been formulated: What is the effect of prolonged GCBT compared with GST for SAD with APD? Since people included in this trial will be diagnosed with both SAD and APD, improvements can be realized in two different domains: with respect to SAD symptoms and with respect to severity of APD traits. Therefore, the research question can be more explicitly formulated in the following questions: How do the effects of prolonged GCBT and GST compare for social anxiety disorder? and How do the effects of prolonged GCBT and GST compare for avoidant personality disorder?

Investigating predictors, moderators and mediators of treatment can add valuable knowledge to our understanding of for whom, under what conditions and how treatments work, thus generating valuable hypotheses for future research [25]. Therefore, this study will, as a secondary objective, look at possible predictors, moderators and mediators of changes on the primary outcome measures. As putative mediators, emotion regulation, self-esteem and schema mode manifestations will be repeatedly measured and associated with outcome. To detect possible predictors and moderators of treatment, the (differential) predictive value of different baseline measures for changes on the primary outcome measures will be explored.

## Methods/design

### Design

**Fig. 1:**
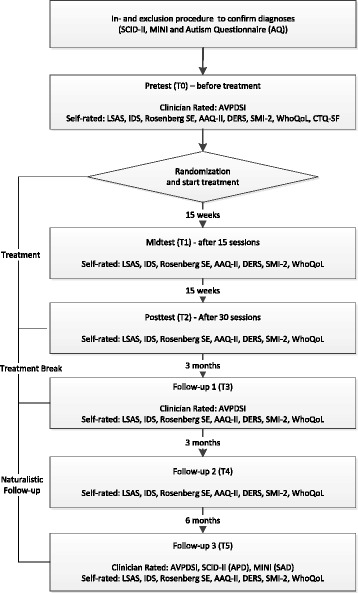
Flow chart of enrolment, intervention and assessments

The design of the study will be a 30-session (on a weekly basis), two-group (GST, GCBT) randomized controlled clinical trial with repeated measurements at baseline (T0), mid-test (T1), post-test (T2) and at 3 months follow-up (T3), half-year follow-up (T4) and 1-year follow-up (T5). Assessment will include diagnostic interviews, symptom questionnaires and quality of life, self-esteem, schema-related and emotion regulation measures. See Fig. 1 (Flow chart of enrolment, intervention and assessments) and Additional file 1 (Standard Protocol Items: Recommendations for Interventional Trials (SPIRIT) flow diagram) for an overview of the study; the SPIRIT checklist is presented in Additional file 2. Diagnostic interviews are based on the DSM-IV classification system [2], since the DSM-5 [8] was not yet available during the developmental phase of this study and there was an absence of diagnostic instruments based on the DSM-5 at the start of the study.

After conclusion of the experimental part of the study (3 months after the last session of the treatment), patients will enter a naturalistic follow-up period in which they are allowed to seek help the way they would normally do when they feel in need for further treatment.

The study will be performed at two sites of PsyQ, a large ambulatory mental health organization in the Netherlands. Two departments of anxiety disorders of PsyQ will participate, one located in The Hague and the other in Rotterdam. Other departments, for instance, the departments of depression or personality disorders, in the regions of The Hague and Rotterdam will be informed of the study and will be asked to refer eligible patients. If necessary to guarantee a sufficient inclusion of eligible patients, more treatment centres will be approached for study participation.

### Population/sample size

Before the start of the randomized controlled trial (RCT), a chart review showed that both the PsyQ location of Rotterdam and the PsyQ location of The Hague have an annual patient flow of 40 patients with both SAD as well as a comorbid APD. This represents an overall yearly *N* of 80, of which it is expected that approximately 90 % (*N* = 72) will be included. This means that every year in each department 36 patients can be randomized over the two conditions. CBT and ST groups consist of a maximum of 9 patients.

We know that CBT has a large effect on SAD compared to waitlist and a small to moderate effect compared to placebo [15]. With respect to ST, in a randomized trial comparing ST, clarification therapy and treatment as usual (TAU), Bamelis et al. [19] found an odds ratio between ST and TAU in recovery from PD diagnosis in the 3–4 range (depending on the specific (sensitivity) analysis), which is equivalent to Cohen’s *d* = .60–.76 [26]. Though a substantial number of patients in the ST condition had a diagnosis of APD, only a small minority of patients received CBT as TAU. No studies are available directly comparing ST with CBT with respect to APD with comorbid SAD. We therefore designed our RCT as a superiority trial with enough statistical power to detect a difference in outcome between treatments (if present) with a medium effect size (Cohen’s *d* = .5). We chose this minimum difference because such a difference is important based on a patient’s perspective or clinical knowledge. Expecting larger differences in outcome does not seem realistic and might result in an underpowered study, while detecting smaller differences is of less relevance for clinical practice. Thus, we designed our study to detect a medium effect size (0.50) with a power of 80 % and a two-tailed alpha set at 0.05 on the primary outcome measures, severity of social anxiety (Liebowitz Social Anxiety Scale Self-Report, LSAS-SR) and severity of avoidant personality disorder (Avoidant Personality Disorder Severity Index, AVPDSI). This implies that 64 patients per study group and in total 128 patients are needed for the present project.

### Inclusion and exclusion criteria

Patients aged between 18 and 65 with primary diagnoses of SAD on Axis I and comorbid APD on Axis II will be included in the study. Primary diagnosis is defined as the diagnosis on which treatment should focus at first instance according to both patient and clinical staff. Other inclusion criteria are willingness, motivation and practical ability to attend 30 sessions of group therapy and to refrain from treatment or counselling outside the context of the present trial. The use of antidepressant medication or benzodiazepine will be permitted, under the condition that the medication dose has been stable for at least 3 months before inclusion and patients are willing not to raise the dosage of the medication during the active phase of the trial. Furthermore, patients must be willing to complete homework assignments between sessions and must sign written informed consent to participate in the study.

Exclusion criteria are suicidality and the presence of psychotic or bipolar symptoms, because these conditions could interfere with the measurement and treatment procedures and suggest an immediate need for alternative interventions. Another exclusion criterion is an Axis I diagnosis of substance abuse or dependence which needs, according to the clinical staff, detoxification (after successful detoxification patients can participate). Moreover, patients with a primary Axis II diagnosis of a borderline, antisocial, schizoid, schizotypal personality disorder will be excluded, because they need highly specialized treatment. Patients in whom there is a suspicion of an autism spectrum disorder (based on a sum score on the Autism-Spectrum Quotient (AQ) higher than 32) [27], who have an IQ less than 80 (in case of suspicion an intelligence test is taken), and problems with the Dutch language (talking, reading, writing) will also be excluded.

### Inclusion and exclusion procedure and randomization

Patients who are registered for treatment at the department of anxiety disorders will be screened on the presence of Axis I disorders by the intake clinician. Eligible patients are patients diagnosed with SAD, or in the case of more than one Axis I diagnosis (e.g. panic disorder or depressive disorder), patients for whom social anxiety should be the principal focus of treatment, according to both patient and clinical staff. At first instance these patients will receive concise information about the study by the intake clinician. At PsyQ The Hague they will then receive a leaflet containing information about the study, which is sent by mail after a research assistant has informed the patient by phone. At PsyQ Rotterdam the leaflet is handed over at the first appointment by the intake clinician or during a second appointment in which the patient is given treatment advice. Patients of that location will be contacted by the research staff after the Axis II diagnosis of APD is established.

Eligible patients are invited for a diagnostic interview — consisting of the Structured Clinical Interview for DSM-IV Axis II Personality Disorders (SCID-II) — to determine the presence of Axis II disorders and other inclusion and exclusion criteria. Beforehand patients receive the AQ and the pre-screener of the SCID-II. During the SCID-II interview all cluster C personality disorders are fully investigated. All confirmatively answered and all unanswered questions on the pre-screener will also be assessed with the SCID-II interview. If the personality trait is confirmed and the amount of confirmed traits pertaining to a certain personality disorder exceeds three, all traits pertaining to that personality disorder will be assessed with the SCID-II interview. Besides scores on the pre-screener, clinical judgement can also be a reason to fully examine a certain personality disorder. In addition to SCID-II, the Mini-International Neuropsychiatric Interview (MINI), section social anxiety, is also administered. The MINI interview is part of the intake procedure of both anxiety departments of PsyQ in addition to a general clinical interview. Some of the patients entering this study will have recently signed up for treatment and will have completed this procedure, but referral by other departments or during ongoing treatment is also possible. It was therefore decided to add the SAD section of the MINI to ensure that patients included meet the inclusion criteria of both SAD and APD.

During the examination, patients will receive additional verbal and extensive written information about the study with respect to both treatment conditions and consequences of participating. After conclusion of the SCID-II administration, patients will be either informed directly or receive a follow-up appointment to discuss the findings of the interview. Patients will have 2 weeks to consider participation, after which they will be contacted by a research assistant. Written informed consent will be obtained from patients who are willing to participate, and they will be asked to fill in the baseline assessment comprising different self-report questionnaires. To be able to assess the frequency and severity of manifestations of APD, a semi-structured interview was developed: the AVPDSI. The AVPDSI interview is administered by phone by a trained psychologist or a trained master student in clinical psychology who fulfils a traineeship at the PsyQ anxiety department.

After a final file check of inclusion and exclusion criteria based on the patient file and after all baseline measures are completed, a research assistant will randomize the patients to the GCBT or GST condition using a computer-generated list (https://www.random.org/lists/). Patients will be randomized in pairs or multiples of pairs. In case of uneven dropout from treatments, a weighted randomization will be done to prevent one of the groups from becoming too small. For both locations this will be done separately.

The main researchers and other outcome adjudicators are blind to group assignment to protect integrity of data collection and analysis. Because it is impossible for a therapist to offer and for a patient to receive therapy without being aware of the techniques they are using or receiving, therapists and patients are not blind. Finally, because data collectors are also employed as therapists in the anxiety departments of the participating sites, they are able to access patient records. Therefore, data collection of clinician-administered measures cannot be blinded. To reduce the risk of biased data collection, therapists involved in delivering either the GCBT or GST are not involved in data collection.

### Interventions

Different guidelines recommend CBT for treatment of SAD [28–30]. CBT is seen as the gold standard for SAD with respect to non-pharmacological treatment, and gains achieved with CBT seem to persist longer than those achieved with pharmacotherapy [29]. Guidelines differ with respect to the advised treatment format (individual or group therapy). The Canadian guidelines do not express any preference, NICE guidelines advise individual CBT and Dutch guidelines recommend group CBT [28–30]. None of these guidelines advises on the treatment of SAD and comorbid APD. With respect to APD, guidelines are scarce. Dutch multidisciplinary guidelines recommend group behavioral treatment for patients with APD. They specify that in case of comorbidity of SAD and APD, CBT is seen as an adequate treatment option, although prolonged treatment is necessary to realize comparable favourable outcomes as for SAD only [13].

GCBT is a regular treatment option and fits neatly in the health care program offered at the RCT participating departments of anxiety disorders. GST will be a new treatment, but will be performed by skilled and experienced therapists. Both treatments were piloted in advance to enable therapists to become acquainted with the treatment protocol and the semi-open group format. In the ST condition therapists had no former experience with ST in a group format; therefore, this treatment modality was permitted an 8 months longer pilot period. In The Hague the pilot period for GST was 18 months and for GCBT 10 months. In Rotterdam the pilot period for GST took 32 months and for GCBT 24 months, this was due to a major reorganisation. 

For patients of both GST and GCBT, treatment comprises of 30 weekly group sessions of 90 minutes, preceded by 2 introductory individual sessions of 45 minutes. In addition to the group treatment, limited individual time with the therapist is permitted with a maximum of 180 minutes. Individual contact can take place in person or by phone or e-mail and can be initiated by either patient or therapist, for instance if urgent personal matters come up or if the therapist fears dropout and wants to discuss possible obstacles for the patient. Sessions will be audiotaped to assess protocol adherence. For both GST and GCBT, customized rating forms will be developed and a random selection of tapes will be listened to in order to evaluate to what extent therapists adhere to the protocol.

### Group schema therapy (GST)

GST is a recently developed treatment for personality disorders. GST is shorter, more directive, confrontational and integrative — using experiential, cognitive and behavioral techniques, addressing childhood and present — than traditional psychodynamic psychotherapies for personality disorders. Schemas can be described as psychological constructs that include beliefs that we have about ourselves, the world and other people, which result from interactions of innate temperament and early environmental factors, notably trauma and unmet core childhood needs. When maladaptive schemas are triggered, specific states occur, as the result of the combination of activated schema(s) and type of coping, that can be described as schema modes. Schema modes are central in GST. Schema modes often had an adaptive role in childhood (e.g. in terms of survival in an adverse situation), but become maladaptive and limiting in adulthood. GST aims at reducing the use of dysfunctional coping modes, healing the maladaptive child modes, banishing the internalized parent modes and strengthening the adaptive modes (healthy adult mode and happy child mode). The ultimate goal of GST is to enable patients to get their emotional needs met, including gaining autonomy and forming healthy social relationships. In GST the group is used as an analogue for the family of origin with the other group members as ’siblings’ and the therapists as ’parents’. Group processes can be used to create corrective emotional experiences and to reduce maladaptive coping modes [24].

For this study, a 30-session treatment protocol of 90-minute weekly sessions over 9 months was developed. The treatment protocol is naturalistic in the sense that, apart from the framework directing treatment focus throughout sessions, therapists do not have a session-to session detailed treatment manual. The structure of the group will be semi-open, and every 10 weeks new patients can enter an ongoing group. Before a patient enters the group, two individual meetings will be planned to provide some information about the content and the process of GST, to discuss briefly the results of the Schema Mode Inventory (SMI) and to formulate a case conceptualization in schema mode terms in collaboration with the patient.

Participants are encouraged to engage in homework between sessions to gain corrective experiences in line with individual needs. Thus, homework does not focus on exposure to social situations that are feared, but on finding ways to better meet one’s needs. Each course will be provided by two experienced therapists who received formal GST training by Farrell and Shaw. They will receive monthly supervision (1.5–2 hours) by Farrell and Shaw using Skype.

### Group cognitive behavioral therapy (GCBT)

GCBT will follow a prolonged version of the well-investigated protocol of Heimberg and colleagues of group therapy for SAD [31–33] in combination with some new insights in the working mechanisms of exposure in vivo [34]. GCBT will mainly consist of simulated exposures to feared situations in-session, cognitive restucturing and in vivo exposure to feared social situations between sessions. Just as in the GST, the format of the group will be semi-open, allowing new patients to enter the group every 10 weeks, and the group treatment of 30 weekly sessions is preceded by 2 individual sessions.

The individual sessions are aimed at facilitating the start of the group treatment. During these sessions information about the content of the therapy is provided and an individual case conceptualization is drawn. Emphasis will be placed on the role of avoidance and safety-seeking behaviors in the maintenance of SAD. The basics of cognitive restructuring will be presented, and patients will be asked to write down a list of feared situations which will be placed in a hierarchical sequence. Social anxiety will be considered as a learned response to social situations incorporating interactive physiological, behavioral and cognitive features. Social anxiety disordered patients supposedly fear most social situations because of their expectancies to be humiliated or rejected by others or confrontations with their own supposed social inadequacies and failure. The main therapeutic target in GCBT is the disconfirmation of these dysfunctional expectancies. To a small extent one strives for these disconfirmations using traditional cognitive procedures such as identification, intellectual challenging and (possibly) correcting of dysfunctional opinions. By far the largest part of the CBT procedure of disconfirming such expectancies exists of exposure in vivo. Since the understanding of the working mechanisms of exposure in vivo has changed in recent years, the main target of exposure in vivo is no longer habituation of the fear response. Nowadays it is believed that exposure in vivo sets off a process of inhibition in which the association of the conditioned stimulus (CS) (social situations) and the feared unconditioned stimulus (US) (humiliation) is inhibited by the forming of new associations around the CS, of which the association with ‘no-US’ (no rejection) is probably the most important [34]. During group sessions and for homework, patients expose themselves deliberately to the social situations they fear in order to let new non-anxious associations form and to experience in particular that the negative consequences they expected in advance did not become true in reality. Noticing the differences between the expected and the actual outcomes of these confrontations is an important subject for therapeutic discussion after exposure. Therefore, the GCBT protocol is a mix of the well-proven approach of Heimberg and some new insights in the mechanisms of exposure in vivo.

Most group sessions will focus mainly on performance-based gradual and systematic exposure with elimination of the accompanying safety behaviors. Self-exposure to situations that resemble the exposure practiced in-session is prescribed as homework to further promote correction of negative expectancies. GCBT is highly structured, with each session following the same agenda including discussing homework assignments, in-session performance-based exposure and assigning new homework. The group is used to provide an immediate exposure setting while it offers the possibility of vicarious learning from other group members at the same time. The group is, however, mainly disorder-specific in a sense that the primary goal is reduction of social anxiety symptoms. In contrast to GST group processes, childhood experiences and experiential exercises will not be used to promote symptom reduction. Each course will be provided by two experienced cognitive behavioral therapists who have been trained in the specific GCBT protocol. During the trial the therapists will receive supervision every 2 weeks (0.45 to 1 hour) by a licensed CBT supervisor.

### Dropout and follow-up

In case of emergencies (like severe increase of symptoms, suicidality) deviation from the protocol is allowed. Subjects can leave the study at any time for any reason if they wish to do so. The researcher can — in consultation with the group therapists — also decide to withdraw a subject from the study for urgent medical or psychological reasons. Reasons for dropout or withdrawal will be registered. Patients who withdraw from treatment will also be asked to complete follow-up assessments. As mentioned before, during the active treatment phase, participation in other treatments, starting medication or raising the medication dosage is not allowed. Also missing too many sessions is not permitted (patients should at least attend a minimum of 80 % of the sessions); if too many are missed, treatment will be terminated (pushout). In consultation with the patient, taking into account the reason for dropout, a suitable alternative will be discussed.

The follow-up period is experimental for the first 3 months (patients are not provided additional treatment unless clinically deemed necessary, e.g. in case of crisis). After this period, patients are offered an appointment with one of the group therapists. Depending on the wish of the patient, main treatment results and further treatment needs can be discussed. From then on follow-up will be naturalistic, meaning that patients are free to seek help in case of unsatisfactory treatment results and/or additional need for treatment.

### Instruments

The measures described below will be administered on different measurement occasions. At all time points self-report measures will be conducted online, except for the AQ and pre-screener of the SCID-II used in the inclusion and exclusion procedure (see inclusion procedure above). On request, participants can complete the self-report measures on paper.

The administration of the SCID-II and the MINI during the intake phase will be conducted face to face. At the last assessment (T5) patients can choose between a face-to-face or telephone administration of the SCID-II, APD section and the MINI, SAD section, according to their preference. For practical reasons, all AVPDSI assessments are administered by phone. Research supports the validity of structured telephone interviews for data collection from primary care patients [35]. See Fig. 1 for the exact measurements per occasion. To ensure privacy of participants and safe data storage, data will be entered in a data file anonymously; participants will be given a unique number, and only the research assistant will be able to link the number with the name of the patient. Data will be stored on a laptop used only for research purposes, and a salted and encrypted file server which has been advised by an independent tech consultant.

### Primary outcome measures

The primary outcome measure for severity of social anxiety is the *self-report version of the Liebowitz Social Anxiety Scale (LSAS-SR).* The objective of the LSAS [36] is to assess the range of social interaction and performance in situations which patients with SAD may fear. The scale contains two parts: one part measures the level of anxiety, and the other part measures the level of avoidance. It consists of 24 items, 13 relating to performance anxiety and 11 concerning anxiety in social situations [37]. The LSAS was originally developed as a clinician-administered semi-structured interview. The LSAS self-report version has comparably good psychometric properties (convergent and discriminant validity, internal consistency, test-retest reliability, criterion validity), high correlation with the clinician-administered version and sensitivity to treatment change. Furthermore, the self-report version is easier to administer and is less time-consuming for both patient and clinician [38–40]. The LSAS-SR will be administered at all time points, i.e. at the start, halfway and at the end of the treatment period and during follow-up at 3, 6 and 12 months after study treatment (see Fig. 1).

The primary outcome measure for severity of APD is *the clinician-administered Avoidant Personality Disorder Severity Index (AVPDSI).* The AVPDSI is a semi-structured interview developed specifically for this study to assess the frequency and severity of manifestations of APD, as defined in the DSM-IV/5, during the last month. The purpose of this instrument is to yield a quantitative index of the current severity of APD manifestations, in terms of both avoidance and fear. The AVPDSI is modelled after the Borderline Personality Disorder Severity Index (BPDSI), which was originally developed by Weaver and Clum [41] and further developed and validated in Dutch by Arntz et al. [42] and Giesen-Bloo et al. [43]. The information gathered by the AVPDSI in the context of the RCT will also be used in a larger study in which the psychometric properties of the interview will be assessed. To ensure quality of data collection of this newly developed instrument, a training protocol will be developed and all measurements will be audiotaped. A random selection of tapes will be listened to in order to assess interrater reliability. This semi-structured interview will be conducted before the start of treatment and at 3 and 12 months follow-up (see Fig. 1).

### Secondary study parameters

#### Mini-International Neuropsychiatric Interview (MINI), social anxiety disorder section

The presence of SAD will be assessed with this section of the MINI [44, 45] as a part of the inclusion procedure and at 12 months follow-up (see Fig. 1). The validation of the Dutch translation of the clinician rated (CR) version of the MINI [46] against the Structured Clinical Interview DSM-III-R-patient version (SCID-P) and the Composite International Diagnostic Interview for ICD-10 (CIDI) showed good to very good kappa values [45].

#### Structured Clinical Interview for DSM Axis II Disorders (SCID-II), APD section

The APD section of SCID-II will be used to assess the presence of APD during inclusion and 12 months after treatment (see Fig. 1). Studies found adequate to good interrater reliability for SCID-II interviews [47–49].

#### Schema Mode Inventory 2 (SMI-2)

The SMI-2 [23] is a modified version of the SMI-1 [50] and successfully distinguishes between patients and controls. Newly formulated modes proved to be appropriate for histrionic, avoidant and dependent personality disorder. The results supported recent theoretical developments in schema therapy (ST). The questionnaire is useful for application in clinical practice. Questions from the SMI-1 [50] concerning the happy child mode were added to the SMI-2 because of its putative therapeutic relevance in the ST treatment for patients with APD.

#### Inventory of Depressive Symptomatology Self-Report (IDS-SR)

Depressive symptomatology is measured by the IDS-SR [51], which encompasses all the relevant symptoms for major depressive disorder based on the DSM-IV [2]. It consists of 30 items, with total scores ranging from 0–84 (only 28 of the 30 items are rated, with each item rated 0–3) [52]. The questionnaire has highly acceptable psychometric properties with reasonable internal consistency, interrater reliability and concurrent and discriminant validity and is a sensitive measure of symptom severity in depression [51, 53]. The self-report version is as sensitive to change as the clinician-administered version [52].

#### World Health Organization Quality of Life–BREF (WHOQOL-BREF)

The WHOQOL-BREF [54] was developed as an international cross-culturally comparable self-report quality of life assessment instrument. It assesses the individual’s perceptions of quality of life in the context of their culture and value systems, personal goals, standards and concerns across four domains: physical health, psychological health, social relationships and environment. The internal consistency of the four domains of the WHOQOL-BREF ranged from 0.66 to 0.80. Domain scores of the WHOQOL-BREF correlated around 0.92 with the WHOQOL-100 domain scores. Relatively low correlations were found between demographic characteristics (age and sex) and WHOQOL-BREF domain scores. It is concluded that the content validity, construct validity and the reliability of the WHOQOL-BREF in a population of adult Dutch psychiatric outpatients are good [54].

#### Difficulties in Emotion Regulation Scale (DERS)

The DERS [55] is a 36-item self-report questionnaire consisting of six subscales: (1) Non-acceptance of emotional responses (6 items); (2) Difficulties engaging in goal-directed behavior (5 items); (3) Impulse control difficulties (6 items); (4) Lack of emotional awareness (6 items); (5) Limited access to emotion regulation strategies (8 items); (6) Lack of emotional clarity (5 items). The internal consistency for the total scale as well as for the subscales appeared to be adequate (varying between a Cronbach’s alpha of 0.80 and 0.93). The questionnaire also has adequate construct and predictive validity and good test-retest reliability.

#### The Rosenberg Self-Esteem Scale (RSES)

The RSES will be used to assess self-esteem. It is a widely used 10-item Likert scale self-esteem measure. Items are answered on a 4-point scale — from strongly agree to strongly disagree — measuring positive and negative feelings towards the self [56]. The Dutch version of the RSES is found to be a one-dimensional scale with high internal consistency and congruent validity and a Cronbach’s alpha of 0.89 [57].

#### Acceptance and Action Questionnaire (AAQ-II)

The AAQ-II [58] is a self-report measure designed to assess experiential avoidance. It consists of 10 items which are scored on a Likert scale from 1 to 7 with total scores that vary from 10 to 70. Higher scores are indicative of more acceptance and less experiential avoidance. It appears to be a unidimensional measure with sound factor structure and good reliability and validity. The mean alpha coefficient is .84 and the 3- and 12-month test-retest reliability scores are .81 and .79 respectively [58]. Construct validity, internal consistency, convergent and divergent validity of the Dutch version are comparable with the English version [59].

#### Childhood Trauma Questionnaire-Short Form (CTQ-SF)

The aforementioned questionnaires SMI-2, IDS-SR, WHOQOL-BREF, DERS, RSES and AAQ-II are conducted at all time points (see Fig. 1). In addition to outcome measures, information on sociodemographic and clinical history variables will be collected at baseline. To assess childhood trauma, the CTQ short form [60] is chosen. The short form was developed from the original version of the CTQ which has 70 items [61]. Because of its brevity of administration and assessment of multiple types of maltreatment, it is well suited for treatment studies like this RCT. The short form consists of 28 items measuring physical, sexual and emotional abuse and physical and emotional neglect, and its reliability and criterion-related validity have been established [60]. A recent study in the Netherlands among a large sample of participants (2308) with or without a current depressive and/or anxiety disorder concludes that the five-factor model fits the data well, that the total scores represent a broad dimension of maltreatment during childhood and that the factorial validity and reliability data make it a good instrument for various forms of abuse and neglect in childhood in clinical settings [62].

### Screening instruments

In the inclusion procedure the MINI and the SCID-II, which are both described above, are used to assess respectively Axis I and II disorders. In addition, patients are screened with the Autism-Spectrum Quotient (AQ).

#### Autism-Spectrum Quotient (AQ)

Since the presence of an autism spectrum disorder (ASD) requests a highly specific treatment and patients with an ASD would probably not benefit from the treatments offered in the RCT, it was decided to exclude patients with a score exceeding the clinical cut-off score on the Autism-Spectrum Quotient (AQ). Patients who score above the cut-off are offered referral for full diagnostic assessment. The AQ is a short self-administered scale for identifying the degree to which any individual adult of normal IQ may have autistic traits. Individuals score in the range 0–50. The higher the score, the more autistic traits a person possesses [27, 63] with a cut-off of 32 for distinguishing individuals with clinically significant levels of autistic traits [27]. The AQ comprises 50 questions assessing five different areas: social skill, attention switch, attention to detail, communication and imagination. It has reasonable construct and face validity and good test-retest and interrater reliability [27] and is a useful screening instrument in clinical practice [64]. The Dutch translation has been shown to be a reliable instrument to assess autism spectrum condition (ASC) traits and has satisfactory internal consistency and test-retest reliability. High total AQ scores are specific to patients with an ASD and set them apart from both the general population and from patients with SAD [65].

### Statistical analysis

Pre-test differences between both treatment groups and between treatment completers and dropouts will be calculated by means of independent *t* tests, nonparametric tests or chi-square tests when appropriate. Multilevel analysis (MLA) will be used to investigate the effectiveness of GCBT and GST in reducing social anxiety symptoms and manifestations of AVPD, and the secondary outcomes. MLA is especially suitable to analyse repeated measure data because it takes into account the dependencies among observations nested within individuals. Another advantage of this methodology is its ability to handle missing data, a problem often occurring in longitudinal research [66]. The data has a three-level hierarchical (multilevel) structure: repeated measures at the first level, individuals at the second level, treatment condition at the third level. For dimensional outcomes the appropriate underlying distribution (e.g. normal, gamma, negative binomial) will be determined based on the observed distributions of residuals. For the repeated part, the best fitting covariance structure will be determined empirically. Moreover, multilevel logistic regression analysis will be performed in order to investigate whether GCBT compared to GST results in a higher proportion of remitted APD. Survival analysis will be used to compare conditions in treatment retention. The identification of possible predictors, moderators and mediators will be based upon the recommendations of Kraemer [25]. Linear regression methods will be applied to identify predictors/moderators related to treatment outcome. Bootstrapping methods [67] will be used to investigate the mediating effects of, for example, emotion regulation on symptom reduction. Furthermore, effect sizes and clinical significant change according to the Jacobson and Truax criteria [68] will be calculated to estimate respectively the magnitude of the treatment effect and the significance of the results for clinical practice.

## Discussion

This article describes the study protocol of an RCT comparing group CBT and group ST for patients with SAD and comorbid APD. Evidence of the effectiveness of group ST on PDs other than borderline personality disorder (BPD) is sparse [69], and to the best of our knowledge, this is the first study on group ST for this specific patient group. Therefore, this trial can provide valuable information on the effectiveness of ST offered in a group format as a treatment option for these patients. It will also extend our knowledge on prolonged CBT, which is the advised treatment for patients with APD according to the Dutch treatment guidelines for treatment of personality disorders [13].

Research on the optimal treatment of APD is scarce. Most studies have investigated treatment of SAD and compared the outcome of patients with and without comorbid APD [70]. APD is in general relatively understudied [71], although it is one of the most prevalent personality disorders across community and clinical samples and is known by its significant functional impairment [71–73]. Since APD is often comorbid with SAD, with data on comorbidity varying between 32–50 % [3], this study can provide clinically relevant information for a substantial group of patients with APD.

The current study has several strengths. The chosen design, an RCT, is recognized to be the best and most definitive way of demonstrating that an intervention is effective [74]. Both treatments are well documented [17, 24, 75] and have been implemented in scientific research before in treatment studies for GCBT (e.g. [76–79]) and individual and group ST (e.g. [19, 43, 80, 81]).

Treatments will be provided by therapists with ample experience in anxiety treatment in specialized mental health care. Besides being trained in the treatment protocol, therapists will be supervised by experienced CBT and ST supervisors.

Both treatment protocols will be suitable for use in a clinical setting. The semi-open character of the groups, in which influx of patients is allowed every 10 weeks, diminishes waiting time and is therefore an ethical solution in case of long waiting lists as a consequence of lengthy treatments for patients with severe psychopathology. Also, from an organizational perspective, in which limitation of waiting time is considered important, this format can be attractive and might possibly even offer a cost-effective option for treatment of APD [13]. The semi-open character enables formation of groups with comparable psychiatric morbidity, allowing for contact with fellow patients. The size of the intended study sample will be sufficient to reliably detect differential treatment effects of a medium effect size or larger, and the relatively limited waiting time is expected to have a positive effect on the influx of patients. The selected measuring instruments are scientifically sound, and the repeated measurements allow for a close monitoring of changes in time, as well as mediation analyses of putative working mechanisms. The follow-up period of one year enables us to monitor how patients fare after the study treatment.

However, there are also some limitations to consider, given the chosen research design. The decision to compare GST with GCBT, *with GCBT as treatment as usual (TAU),* instead of a no-treatment or attention control condition might affect internal validity, since it sets boundaries on the possibility to control for other potentially influencing factors not directly related to the therapeutic context. When the effects of both treatments prove to be not significantly different, the absence of an inactive condition implies that one cannot exclude the possibility that factors such as passage of time or attention may have critically influenced outcomes observed. The influence of the factor time is, however, expected to be limited, since APD, like other personality disorders, is by definition assumed to be persistent [8], and recent studies show that APD symptoms are relatively stable over time [71, 82, 83]. Moreover, the addition of a control group would, in our opinion, have some serious ethical consequences, because it would deprive help-seeking patients of treatment for too long a period of time.

Also one might argue the validity — for this specific patient group — of GCBT as TAU, because of the relative paucity of scientific evidence for prolonged CBT for APD. Although we subscribe the need for additional scientific evidence, we feel that prolonged GCBT can be considered as TAU based on Dutch guidelines [13] and earlier studies on SAD with comorbid APD [16] or APD with or without comorbid SAD [84–86].

Another point to consider is that both treatments consist of two individual sessions followed by 30 group sessions. A ST treatment of thirty sessions is relatively short. Most published RCTs investigating ST treatments thus far comprise 50 or more sessions covering time spans of more than one year [19, 81, 87]. A longer duration of treatment might possibly show greater improvements on the outcome measures. On the other hand, the RCT of Farrell et al. [80] compared the effectiveness of adding 30 group sessions of ST to TAU and TAU alone in patients with BPD and found significantly greater reductions in BPD symptoms and global severity of psychiatric symptoms in the GST. So we expect that this treatment period will be long enough to detect differential effects of treatment if present.

The semi-open structure of the group treatments might have disruptive effects on the course of therapy because of patients entering and leaving at several time points. The possible difficulties experienced by the newcomers are addressed by adding two individual sessions and by reframing something that might be frightening into something positive (e.g. for CBT to facilitate exposure, and for ST to provoke dysfunctional modes, which can subsequently be addressed). One cannot rule out that this treatment format might influence effectiveness and that this effect is different for both treatment modalities.

Furthermore, execution of the multicentre RCT in two large cities in the Netherlands and under tightly controlled conditions might have implications for generalizability and external validity. When treatment results are promising, implementation studies of ST under more naturalistic conditions (benchmark studies) are needed to obtain further evidence for the treatment modalities.

In conclusion, this RCT will extend our knowledge on the effectiveness of both GCBT and GST and will give us an opportunity to examine if GST is a valuable option for patients suffering from SAD in combination with APD. Moreover, the results will inform us if the effects of GST exceed those of existing practices mainly consisting of prolonged CBT interventions.

Since people with this double diagnosis report a low quality of life and are seriously impaired in social interactions and fulfilling social relationships with a negative impact on their well-being, developing and testing an additional and easily applicable treatment option will have both scientific, clinical and individual value, taking us a step forward in treating these severely disabled patients.

### Trial status

The trial was registered on 25 March 2013 in the Dutch Trial Register (NTR3921). Participants are currently being recruited and enrolled. The trial started recruitment in March 2014 at PsyQ The Hague and in May 2015 at PsyQ Rotterdam.

## Additional files

Additional file 1: SPIRIT flow diagram. (PDF 143 kb)
Additional file 2: SPIRIT checklist. (PDF 160 kb)

## Abbreviations

AAQ-II: Acceptance and Action Questionnaire
APD: Avoidant personality disorder
AQ: Autism-Spectrum Quotient
ASC: Autism spectrum condition
AVPDSI: Avoidant Personality Disorder Severity Index
BPD: Borderline personality disorder
BPDSI: Borderline Personality Disorder Severity Index
CBT: Cognitive behavioral therapy
CS: Conditioned stimulus
CTQ-SF: Childhood Trauma Questionnaire-Short Form
DERS: Difficulties in Emotion Regulation Scale
GCBT: Group cognitive behavioral therapy
GST: Group schema therapy
IDS-SR: Inventory of Depressive Symptomatology Self-Report
LSAS-SR: Liebowitz Social Anxiety Scale Self-Report
LUMC: Leiden University Medical Centre
MEC: Medical Ethical Committee
MINI: Mini-International Neuropsychiatric Interview
MLA: Multilevel analysis
RCT: Randomized controlled trial
RSES: Rosenberg Self-Esteem Scale
SAD: Social anxiety disorder
SCID-II: Structured Clinical Interview for DSM-IV Axis II Personality Disorders
SMI-2: Schema Mode Inventory 2
ST: Schema therapy
TAU: Treatment as usual
US: Unconditionned stimulus
WHO-QOL-BREF: World Health Organization Quality of Life–BREF
