# Supplementary material for: Group schema therapy versus group cognitive behavioral therapy for social anxiety disorder with comorbid avoidant personality disorder: study protocol for a randomized controlled trial
Source: Trials. 2016 Oct 8;17:487. doi: 10.1186/s13063-016-1605-9 (PMC5055701; doi:10.1186/s13063-016-1605-9)
Supplement: Additional file 1: — SPIRIT flow diagram. (PDF 143 kb) [file 13063_2016_1605_MOESM1_ESM.pdf]

Figure S1. SPIRIT flow diagram: Schedule of enrolment, interventions and assessments

	**STUDY PERIOD**						
	**Enrolment**	**Allocation**	**Post-allocation**				**Close-out**
**TIMEPOINT**	**$-t_1$ & t_0**	**0**	**t_1**	**t_2**	**t_3**	**t_4**	**t_5**
**ENROLMENT:**							
**Eligibility screen**	X						
**Informed consent**	X						
SCID-II	X						
MINI	X						
**AQ**	X						
**Allocation**		X					
**INTERVENTIONS:**							
GCBT			⟷	⟷			
GST			⟷	⟷			
**ASSESSMENTS:**							
Baseline variables	X						
CTQ-SF	X						
LSAS-SR	X		X	X	X	X	X
AVPDSI	X				X		X
IDS-SR	X		X	X	X	X	X
RSES	X		X	X	X	X	X
AAQ-II	X		X	X	X	X	X
DERS	X		X	X	X	X	X
SMI-2	X		X	X	X	X	X
WHOQol-Bref	X		X	X	X	X	X
SCID-II (APD)							X
MINI (SAD)							X

SCID-II Structured Clinical Interview for DSM-IV Axis II Personality Disorders, MINI Mini International Neuropsychiatric Interview, AQ Autism-Spectrum Quotient, GCBT Group Cognitive Behavioral Therapy, GST Group Schematherapy, CTQ-SF Childhood Trauma Questionnaire-Short Form, LSAS-SR Liebowitz Social Anxiety Scale Self-report, AVPDSI Avoidant Personality Disorder Severity Index, IDS-SR Inventory of Depressive Symptomatology Self-report, RSES Rosenberg Self-Esteem Scale, AAQ-II Acceptance and Action Questionnaire, DERS Difficulties in Emotion Regulation Scale, SMI-2 Schema Mode Inventory 2, WHOQol-Bref World Health Organisation Quality of Life-Bref,SCID-II (APD) Structured Clinical Interview for DSM-IV Axis II Personality Disorders section Avoidant Personality Disorder, MINI (SAD) Mini International Neuropsychiatric Interview section Social Anxiety Disorder.
